# Impacts and Pathways of Behavioral Activation on Psychological Distress Among Patients Diagnosed With Esophageal and Gastric Cancer in China: A Randomized Controlled Trial

**DOI:** 10.1002/cam4.70314

**Published:** 2024-10-15

**Authors:** Runze Huang, Anlong Li, Han Ge, Lijun Liu, Ling Cheng, Mingjun Zhang, Huaidong Cheng

**Affiliations:** ^1^ Department of Oncology The Second Affiliated Hospital of Anhui Medical University Hefei Anhui China; ^2^ Anhui Medical University Hefei Anhui China; ^3^ School of Nursing Anhui Medical University Hefei Anhui China; ^4^ Medical Intensive Care Unit The First Affiliated Hospital of Anhui University of Chinese Medicine Hefei Anhui China; ^5^ The Third School of Clinical Medicine Southern Medical University Guangzhou People's Republic of China; ^6^ Department of Oncology Shenzhen Hospital of Southern Medical University Shenzhen Guangdong China

**Keywords:** anxiety, behavioral activation, esophageal and gastric cancer, psychological distress, psychological mechanisms, self‐efficacy

## Abstract

**Objective:**

The objective of this study is to investigate the efficacy of behavioral activation (BA), a novel psychological intervention, in ameliorating psychological distress and anxiety symptoms among patients diagnosed with esophageal and gastric cancer, as well as the mediating role of self‐efficacy between BA and psychological distress.

**Methods:**

A total of 139 patients diagnosed with esophageal and gastric cancer were recruited in China from March 2023 to October 2023 and randomly assigned to either the BA plus care as usual group (BA+CAU group) or the care as usual group (CAU group). Pre‐ and post‐intervention questionnaires, including the Psychological Distress Thermometer (DT), Generalized anxiety disorder 7‐item (GAD‐7) Scale, General Self‐Efficacy Scale (GSES) and the activation subscale of Behavioral Activation for Depression Scale (BADS‐A), were administered.

**Results:**

Generalized estimating equation analyses revealed that, compared to usual care alone, combining BA with usual care significantly ameliorated psychological distress, anxiety as well as improved self‐efficacy and activation. The mediation analysis revealed that self‐efficacy served as a mediator in the relationship between activation and psychological distress.

**Conclusions:**

BA primarily based on telephone or WeChat can not only directly ameliorates psychological distress and anxiety symptoms in patients with esophageal cancer and gastric cancer but also indirectly alleviates psychological distress by enhancing self‐efficacy. The study also demonstrates the potential of BA in cancer patients, a skill that can be effectively acquired by primary care workers without specialized training and implemented more flexible.

**Trial Registration:** NCT06348940

## 1 Introduction

Almost all cancer patients will experience varying degrees of psychological distress due to cancer diagnosis, cancer reactions from treatment methods, and physical symptoms caused by the disease itself [1]. These manifestations range from mild emotional vulnerability, sadness, and more severe negative emotional symptoms such as anxiety, depression, and fear. The prevalence of these psychological distress or specific emotional symptoms in the cancer population is four times higher than that in the general population [2], with approximately 50% of cancer patients exhibit elevated levels of psychological distress [3]. Furthermore, a comprehensive meta‐analysis has demonstrated that anxiety—specific emotional symptoms experienced by individuals with cancer—are linked to both poorer prognosis and increased mortality rates [4].

The high prevalence of psychological distress or specific negative emotional symptoms among cancer patients, as well as their association with adverse prognosis, has garnered significant attention from researchers endeavoring to address these problems. In addition to traditional pharmacological interventions, researchers are focusing on effective psychosocial intervention methods for individuals with cancer, such as cognitive behavioral therapy (CBT) [5], supportive psychotherapy [6], and family and couples therapy [7]. Behavioral activation (BA) has emerged as a brief and effective psychosocial intervention for depression symptoms [8] and other negative emotional symptoms [9] among these therapies. Its approach to addressing negative emotions involves diminishing rumination, avoidance, and other maladaptive behaviors, while enhancing and reinforcing the frequency of pleasant and meaningful adaptive behaviors in daily life [10]. This process enables individuals to transition from Trigger, Response, Avoidance‐Pattern (TRAP) to Trigger, Response, Alternative Coping (TRAC) [10]. In comparison with other psychosocial interventions, BA offers the advantages of simplicity, structured implementation, and increased flexibility [11]. Moreover, it can enhance regulation of one's overt behavior and life, aiding individuals in addressing life challenges, a feature particularly pertinent for cancer patients who frequently confront situations beyond their control [12]. Hence, it is gaining traction among researchers as a means to address negative emotional symptoms in cancer patients recently. For instance, Akechi et al. found that combining BA with problem‐solving therapy (PST) effectively reduced fear of cancer recurrence and depression symptoms among breast cancer survivors [13], while Fernandez‐Rodriguez et al. demonstrated that BA significantly enhanced both the affective state and interpersonal functioning of in lung cancer and breast cancer patients [14]. Given that psychological distress encompasses negative emotional symptoms [1], and anxiety symptoms are a specific component of emotional symptoms, the implementation of BA may prove beneficial in addressing psychological distress and anxiety symptoms among cancer patients.

However, the majority of current research on BA focuses on breast cancer patients and addresses depression and fear of cancer recurrence symptoms among cancer patients. There is a lack of investigation into the psychological distress and specific anxiety symptoms experienced by esophageal and gastric cancer patients. While upper digestive tract cancers (primarily esophageal and gastric cancers) represent the most prevalent malignancies worldwide and account for a significant number of tumor‐related deaths [15]; ~50% of upper digestive tract cancers occur in China according to statistics from the National Cancer Center [16]. Psychological distress and anxiety symptoms are also frequently observed among patients diagnosed with esophageal or gastric cancer. According to reports, 50% of esophageal cancer patients who underwent surgical resection experienced issues related to worry, anxiety, and depression 5 years post‐operation, with 39% reporting feelings of tension [17]. A separate study revealed that 33.6% of patients diagnosed with gastric cancer across all stages experienced significant psychological distress [18]. Distress and (or) emotional symptoms can significantly impact various aspects of their quality of life [19] and even diminish their survival prospects [20]. Unfortunately, psychological distress is frequently disregarded by medical staff [21]. Given the substantial prevalence of esophageal and gastric cancer patients, their psychological distress and specific anxiety symptoms, are notably salient yet have been frequently overlooked. Moreover, BA holds promise for these issues of cancer patients; therefore, this study opted to implement BA for these patients and to consider psychological distress and anxiety symptoms as indicators of therapeutic efficacy.

Moreover, we chose self‐efficacy as another outcome for it is widely believed to be associated with improvements in emotional well‐being [22]. Individuals with higher levels of self‐efficacy exhibit reduced stress levels and are less susceptible to depression [23]. Conversely, those with low self‐efficacy tend to question their own abilities and display diminished motivation toward pursuing their desired objectives, rendering them more prone to experiencing negative emotional symptoms [24]. Similarly, in psychosocial interventions, enhancements in self‐efficacy often exhibit a strong correlation with ameliorations in negative affective states. For instance, Xu et al. discovered that intervention measures based on the information‐motivation‐behavioral skill model can significantly enhance postpartum self‐efficacy among women who have undergone cesarean section, as well as alleviate symptoms such as anxiety and depression [25]. Similarly, Fancourt et al. observed that individuals experiencing bereavement can gradually improve their sense of self‐efficacy by engaging in choir participation and maintaining more stable levels of depression and well‐being [26]. These findings collectively suggest that self‐efficacy may serve as a potential mediator between psychosocial interventions and psychological distress (psychosocial interventions may mitigate psychological distress through the enhancement of self‐efficacy). However, Kim et al. discovered that although senior nursing students who underwent rational emotive behavior therapy (REBT) exhibited a significant enhancement in self‐efficacy, their stress coping strategies did not witness substantial improvement in various aspects [27]. BA shares the general features of psychosocial interventions and emphasizes behavior therapy [28]; meanwhile, the pathways through which it ameliorates psychological distress in cancer populations remains elusive. Does or not self‐efficacy serve as a mediating factor between BA and psychological distress?

In summary, our study aimed to investigate the impact of BA on ameliorating psychological distress and anxiety symptoms in esophageal cancer and gastric cancer patients, as well as the mediating role of self‐efficacy between BA and psychological distress. The primary outcome measure is psychological distress due to its broader and more encompassing nature. Secondary outcomes include anxiety symptoms, self‐efficacy, and activation (which measures the magnitude of BA's effect). Based on these objectives, our scientific questions are:

1. Can BA effectively mitigate psychological distress and anxiety symptoms in patients with esophageal and gastric cancer?
2. Does self‐efficacy mediate the relationship between BA and psychological distress? (meaning “Can BA mitigate psychological distress through the enhancement of self‐efficacy?”)

## 2 Methods

### 2.1 Study Design

This one‐blind, parallel assignment RCT involved two conditions: BA intervention combined with CAU and CAU alone. Baseline data, including psychological distress, anxiety, self‐efficacy, and activation scores, were collected for all participants at the start of the study. Psychological distress, anxiety, self‐efficacy, and activation scores were assessed 1 month (T1, midpoint of the experiment) and 2 months (T2, ending of the experiment) after baseline. The primary endpoint is psychological distress, with secondary endpoints including anxiety, self‐efficacy, and activation.

### 2.2 Participants

From March 2023 to October 2023, a total of 139 esophageal and gastric cancer patients were enrolled in the Department of Oncology, the Second Affiliated Hospital of AMU. Of these, 70 patients were randomly assigned to the BA+CAU group, and 69 patients to the CAU group.

The inclusion criteria for participants were as follows: patients who (1) had to meet the diagnostic criteria for esophageal or gastric cancer through clinical, pathological, and imaging examinations, (2) should have no history of prior psychological intervention, (3) Karnofsky Performance Status (KPS) score should be equal to or greater than 80 points, (4) were required to complete the questionnaire independently or with assistance from others if needed, (5) were consistently admitted to the hospital for tumor treatment, with an average time interval of approximately 1 month between each hospitalization, (6) Psychological Distress Thermometer (DT) score should be equal to or greater than 4 points, and (7) were of legal age, 18 years or older.

Participants were excluded based on the following reasons: patients who (1) with severe intellectual disabilities or other communication difficulties that hindered normal interaction, (2) with an expected survival time of less than 4 months, (3) were Cachexia or severely debilitated, (4) with fractures, serious heart dysfunction, or other severe medical conditions, and (5) with a history of using psychotropic or psychoactive drugs.

### 2.3 Study Procedure

**FIGURE 1:**
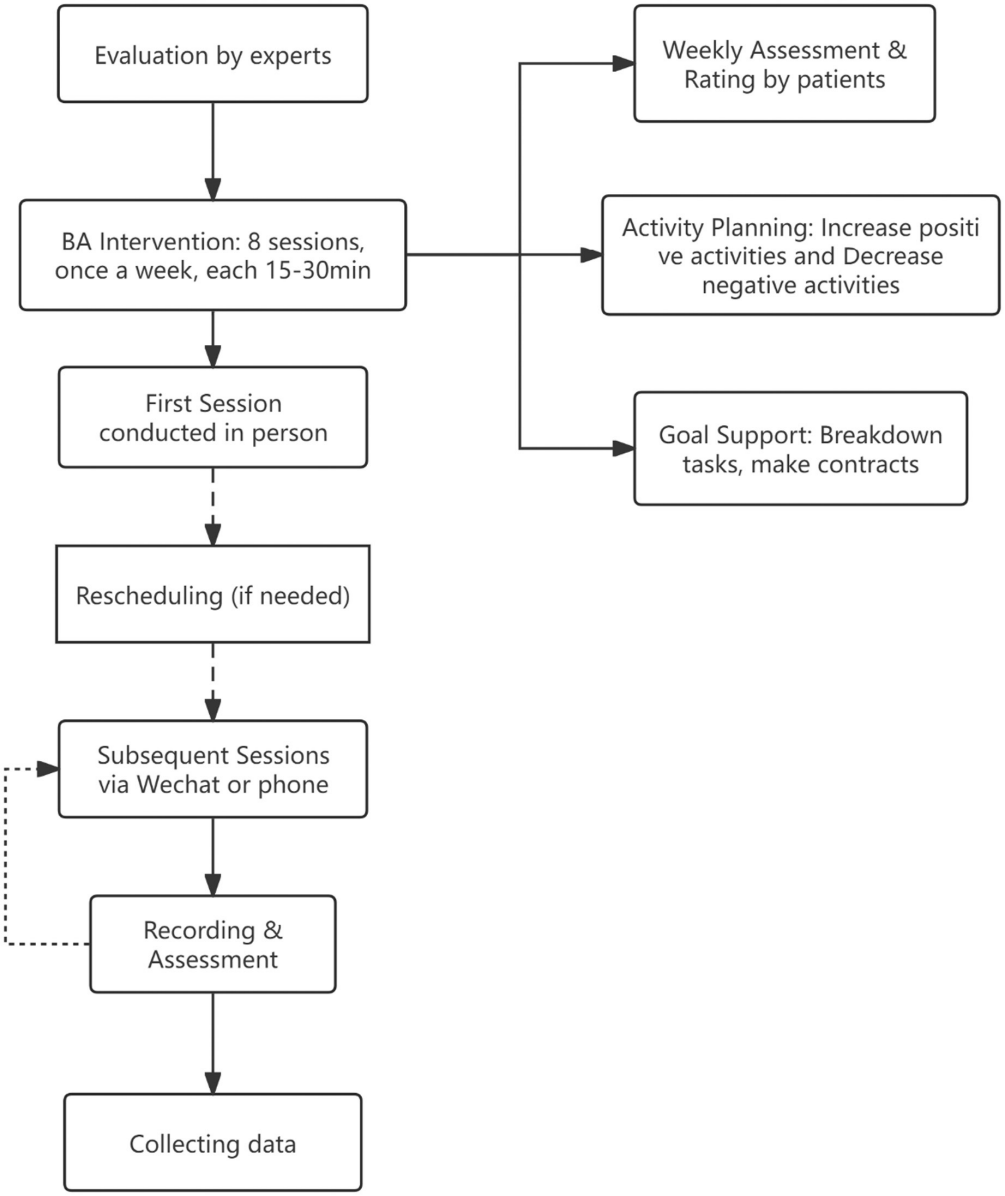
Procedural steps of BA intervention.

This study was evaluated by experts in the field, including two psychologists and one oncologist to ensure its scientificity and feasibility. The BA intervention was administered 8 times, once a week. Each session lasted for 15–30 min. All the interventions were conducted by the same psychological consultant. The first BA intervention for each patient was conducted in person, with a psychological consultant in a separate treatment room. Thereafter, the psychological intervention sessions for most patients were conducted via audio calls or video conferencing means (WeChat or phone). With informed consent from all participants, each audio call was recorded, and these recordings served as the basis for the examiner's assessment of the quality of the intervention. If the BA intervention could not be conducted at the scheduled time due to patients' physical condition or other reasons, it was rescheduled within 3–5 days from the specified intervention time. The procedural steps of the intervention are depicted in Figure 1. In addition to the BA intervention, the BA+CAU group received comparable cancer care and treatment to that of the CAU group. The cancer care and tumor treatment received by patients in the CAU group were standard procedures. Cancer care was exclusively administered by nurses, encompassing daily nursing activities and health education focused on aspects such as lifestyle, dietary habits, and disease prevention. However, it did not incorporate specific interventions to enhance relaxation and pleasure or emotional well‐being among patients. The content of health education provided to each patient remained consistent. Furthermore, the tumor treatment regimen did not encompass any psychosocial interventions.

The aim of BA is to promote engagement in meaningful and pleasurable activities while reducing those that perpetuate negative emotional cycles. We developed a 8‐week program (Data S1) based on the Brief Behavioral Activation Treatment for Depression: Revised Treatment Manual [29]. Patients were assessed weekly and instructed to keep track of their activities from the past week, rating each activity for its level of pleasure and meaning, as well as providing an overall rating for the week. Based on these scores, patients can identify which activities are beneficial or detrimental to their mental health and create a plan to increase positive activities in the following week. If they encounter difficulties achieving these goals, we encourage them to break down larger objectives into smaller specific tasks or make “contracts” with others for support. Additionally, if patients are less active, we suggest selecting some activities from our list (family, social, religious, etc.) as targets.

### 2.4 Randomization

The statistician in the experiment was only responsible for randomization and was not involved in conducting the experiment. After the participants' baseline data was assessed, they were randomly assigned to one of two groups using a computer provided by the statistician. Unbeknownst to all the researchers, the sequence was written on cards and sealed, only to be opened when the groups were to be assigned. We used a single‐blind randomized controlled trial, with the researchers (data collector) being blinded.

### 2.5 Measures

#### 2.5.1 Psychological distress

Psychological distress was measured with the Psychological Distress Thermometer (DT). The DT involves a graphic of a typical thermometer upon which subjects indicate their level of distress. Scores range from 0 to 10, with 0 indicating no distress and 10 indicating extreme distress [1]. A cutoff score ≥ 4 has been shown to perform best in terms of sensitivity and specificity for identifying cancer patients with high psychological distress [30].

#### 2.5.2 Anxiety

Generalized Anxiety Disorder 7‐item scale (GAD‐7), recommended by the NCCN guidelines, was used to assess anxiety disorders in cancer patients. This scale comprises seven items, with each item graded on a four‐point scale: 3 = almost every day; 2 = more than a week; 1 = a few days; and 0 = not at all. The total score is obtained by summing up the scores of all seven items, ranging from 0 to 21. A higher score indicates a greater level of anxiety [31].

#### 2.5.3 Self‐efficacy

Self‐efficacy was measured with General Self‐Efficacy Scale (GSES). The scale consisted of 10 items, with a score for each item of between 1 and 4, and the total score of 10–40, with the final points were divided by 10. Higher scores indicated higher self‐efficacy [32].

#### 2.5.4 Activation

Activation was measured with the activation subscale of Behavioral Activation for Depression Scale (BADS). BADS typically comprises four subscales: Activation, Avoidance/Rumination, Work/School Impairment, and Social Impairment. The activation subscale was utilized to evaluate the impact of BA. It consists of seven items rated on a scale from 0 to 6 (ranging from “not at all” to “completely”), yielding a total score ranging from 0 to 42. Higher scores indicate more favorable outcomes following BA intervention [33].

### 2.6 Sample Size

Due to the dearth of relevant experiments, we utilized data from a pilot study (*n* = 44) to determine the necessary sample size for this study. To calculate the sample size for the randomized controlled trial (RCT), we used PASS software (PASS, version 11). The allocation ratio between the intervention and control groups was 1:1, with a power of *β* = 0.9 and significance level of α = 0.05. For each indicator, the estimated mean difference between the intervention group and control group was found to be 3.431; 6.760; 7.790; 12.590 respectively, while the predicted standard deviation for the control and intervention groups were calculated as 2.182, 1.290; 4.445, 3.471; 6.252, 3.488; 7.330, 5.152. In consideration of a follow‐up loss rate of ~20%, we determined that a minimum sample size of 15; 20; 22; 15 respectively. In addition, for the simple mediation model, we used an online platform to calculate the sample size [34]. The test power *β* was determined to be 0.85, while accounting for a loss to follow‐up rate of 20%. Consequently, we calculated a sample size of 120 participants when psychological distress as the dependent factor in the model. In conclusion, based on the principle of maximizing sample size, this study ultimately included 139 patients, surpassing the minimum requirement of 120 cases.

### 2.7 Statistical Analysis

The statistical analysis in this study was conducted using IBM SPSS Statistics version 22.0. With the exception of the statistical analysis conducted in step 2 below, all other analyses yielded statistically significant results at a significance level of *p* < 0.05. The application of statistical methods is contingent upon meeting all relevant conditions. We managed missing data according to the intention‐to treat (ITT) principle, ensuring that the outcome data analysis accounts for the initial random assignment into the intervention and control groups.

1. Descriptive statistics were utilized to present the demographic data and clinical outcomes of the participants at baseline, which are reported as percentages or mean ± standard deviations (SDs). The balance between the two groups for continuous, categorical and ordinal variables was assessed using independent *t*‐tests, chi‐squared, and rank‐sum tests respectively. If the comparison of rates between the two groups did not meet the conditions for the application of the chi‐squared test, Fisher's exact probability method was used. Additionally, normality for continuous variables was evaluated through the Kolmogorov–Smirnov test.
2. Generalized estimating equation (GEE) models, specifically with an unstructured model, were used to assess the differential change in the outcome variables between the two groups at T1 and T2 compared to T0 for all outcomes. Stratified analyses were conducted as a sensitivity analysis to assess the consistency of the main findings among two subgroups (esophageal cancer and gastric cancer) with the overall population. The *p* values were adjusted using the Bonferroni method due to the performance of stratified analyses. *p* values less than 0.017 (0.05/3) were considered statistically significant, while *p* values ranging from 0.017 to 0.05 were deemed suggestive.
3. Correlation analyses conducted on the differences between T3 and T1 for all observed variables, and subsequently included the significant correlations in the mediation analysis. Based on the results obtained from the correlation analysis and scientific question, we constructed a simple mediation model with activation as the independent variable, self‐efficacy as the mediating variable, and psychological distress as the dependent variable. Following Hayes guidelines [35], SPSS PROCESS macro with the bootstrapping method was applied to assess the mediation model. All socio‐demographic and clinical factors were adjusted in the mediation analyses to overcome potential confounding effects. As the primary tumor site was incorporated and adjusted in our model, we refrained from conducting a stratified analysis of the mediation model.

## 3 Results

### 3.1 Population

**FIGURE 2:**
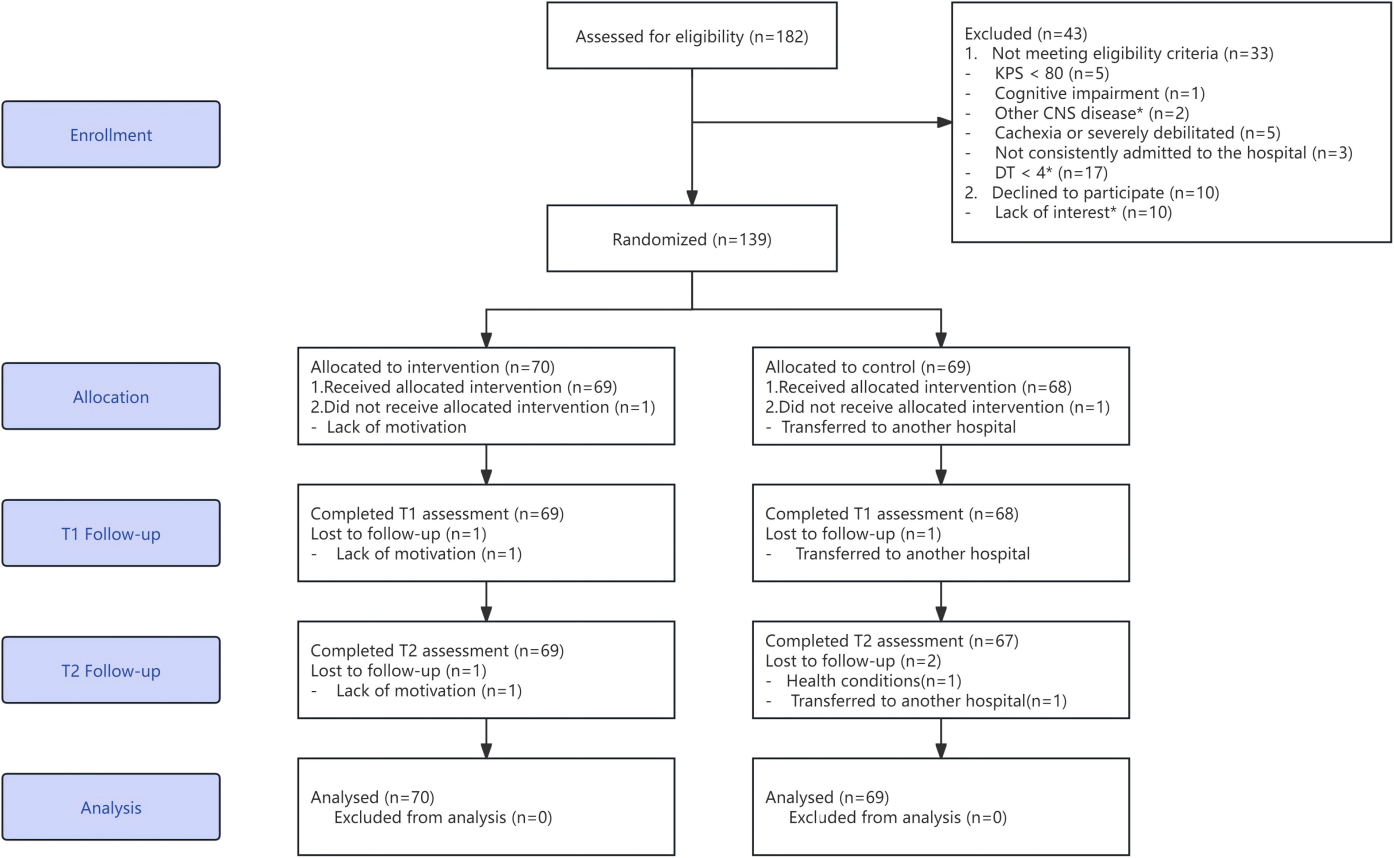
CONSORT flow diagram. *A total of 6 patients were also excluded because they had previously taken psychotropic drugs: Four individuals have ingested sedatives, one has taken mood stabilizers, and one has taken antidepressants.

Figure 2 indicates the CONSORT flow diagram. We screened 182 potential participants, resulting in an enrollment rate of 76.37%, with 139 participants meeting the eligibility criteria and providing consent to participate. These 139 eligible participants were then randomized into two groups: 70 participants were assigned to the BA+CAU group, and 69 participants were assigned to the CAU group. For the BA+CAU group, the mean ± SD, median, and range of completed sessions were 5.8 ± 1.6, 6, and 0–8 respectively. 65 of 70 participants (92.9%) attended at least 4 sessions. The majority of participants engaged in assessments either through hospital readmission or online, with a drop‐out rate of the entire trial was 2.2% (*n* = 3). Among the three dropouts, one in the intervention group withdrew due to lack of motivation (Allocation phase), while two in the control group withdrew due to transferred to another hospital (Allocation phase) and health conditions (T2 phase), respectively.

The participants' baseline socio‐demographic, clinical characteristics and questionnaire scores are summarized in Table 1, which were well balanced. Most of these patients were male, elderly, unemployed or retired, with a middle school education or below, and married. Their primary caregivers post‐admission were their spouses and children. The majority of tumors presented at clinical stage III and IV, with squamous cell carcinoma and adenocarcinoma being the predominant pathological types. 50% or more of the patients received treatment primarily through single chemotherapy or a combination of chemotherapy and immunotherapy. Additionally, approximately half of the patients reported symptoms of depression.

**TABLE 1:** The demographic characteristics and clinical information of the patients (*n* = 139).

Characteristics	BA+CAU (*n* = 70)	CAU (*n* = 69)	*t*/*χ*/*z*	*p*
Gender, *n* (%)			0.790	0.374
Female	12 (17.1)	16 (23.2)		
Male	58 (82.9)	53 (76.8)		
Age (years), mean ± SD	66.7 ± 9.6	65.4 ± 10.5	0.786	0.433
Work, *n* (%)			0.891	0.640
Farmer	5 (7.1)	8 (11.6)		
Others	13 (18.6)	11 (15.9)		
No work or retired	52 (74.3)	50 (72.5)		
Education, *n* (%)			−0.697	0.486
Illiteracy	19 (27.1)	19 (27.5)		
Primary school	25 (35.7)	18 (26.1)		
Middle school	22 (31.4)	27 (39.1)		
College or above	4 (5.7)	5 (7.2)		
Marriage, *n* (%)			0.812	0.367
Unmarried or divorced	5 (7.1)	8 (11.6)		
Married	65 (92.9)	61 (88.4)		
Caregiver, *n* (%)			4.475	0.215
Spouse	19 (27.1)	26 (37.7)		
Children	38 (54.3)	26 (37.7)		
Care of others	5 (7.1)	9 (13.0)		
No care	8 (11.4)	8 (11.6)		
BMI, mean ± SD	20.6 ± 3.2	20.3 ± 3.3	0.608	0.544
Primary tumor site, *n* (%)			0.919	0.631
Esophageal	39 (55.7)	33 (47.8)		
Gastric	24 (34.3)	27 (39.1)		
Gastroesophageal junction	7 (10.0)	9 (13.0)		
Tumor stage, *n* (%)			−0.231	0.817
I	0 (0.0)	3 (4.3)		
II	14 (20.0)	7 (10.1)		
III	13 (18.6)	16 (23.2)		
IV	43 (61.4)	43 (62.3)		
Histology, *n* (%)			0.596	0.742
Squamous carcinoma	34 (48.6)	30 (43.5)		
Adenocarcinoma	31 (44.3)	35 (50.7)		
Others	5 (7.1)	4 (5.8)		
Current treatment, *n* (%)			0.659	0.956
Chemotherapy	16 (22.9)	15 (21.7)		
Radiotherarpy	7 (10.0)	6 (8.7)		
Targeted therapy	3 (4.3)	4 (5.8)		
Chemotherapy + immunotherapy	18 (25.7)	21 (30.4)		
Others	26 (37.1)	23 (33.3)		
Depression symptoms, *n* (%)			0.373	0.541
Yes	32 (45.7)	28 (40.6)		
No	38 (54.3)	41 (59.4)		
KPS, *n* (%)			−0.603	0.547
80	16 (22.9)	18 (26.1)		
90	46 (65.7)	45 (65.2)		
100	8 (11.4)	6 (8.7)		
Questionnaire scores, mean ± SD				
Psychological distress	4.9 ± 1.4	4.5 ± 1.0	1.880	0.062
Anxiety	5.9 ± 4.4	5.2 ± 4.2	0.977	0.330
Self‐efficacy	2.6 ± 0.5	2.6 ± 0.5	0.044	0.965
Activation	28.9 ± 7.7	30.8 ± 7.3	−1.455	0.148

Abbreviations: KPS, Karnofsky performance status; SD, standard deviation.

### 3.2 Intervention Effects on Outcomes

**FIGURE 3:**
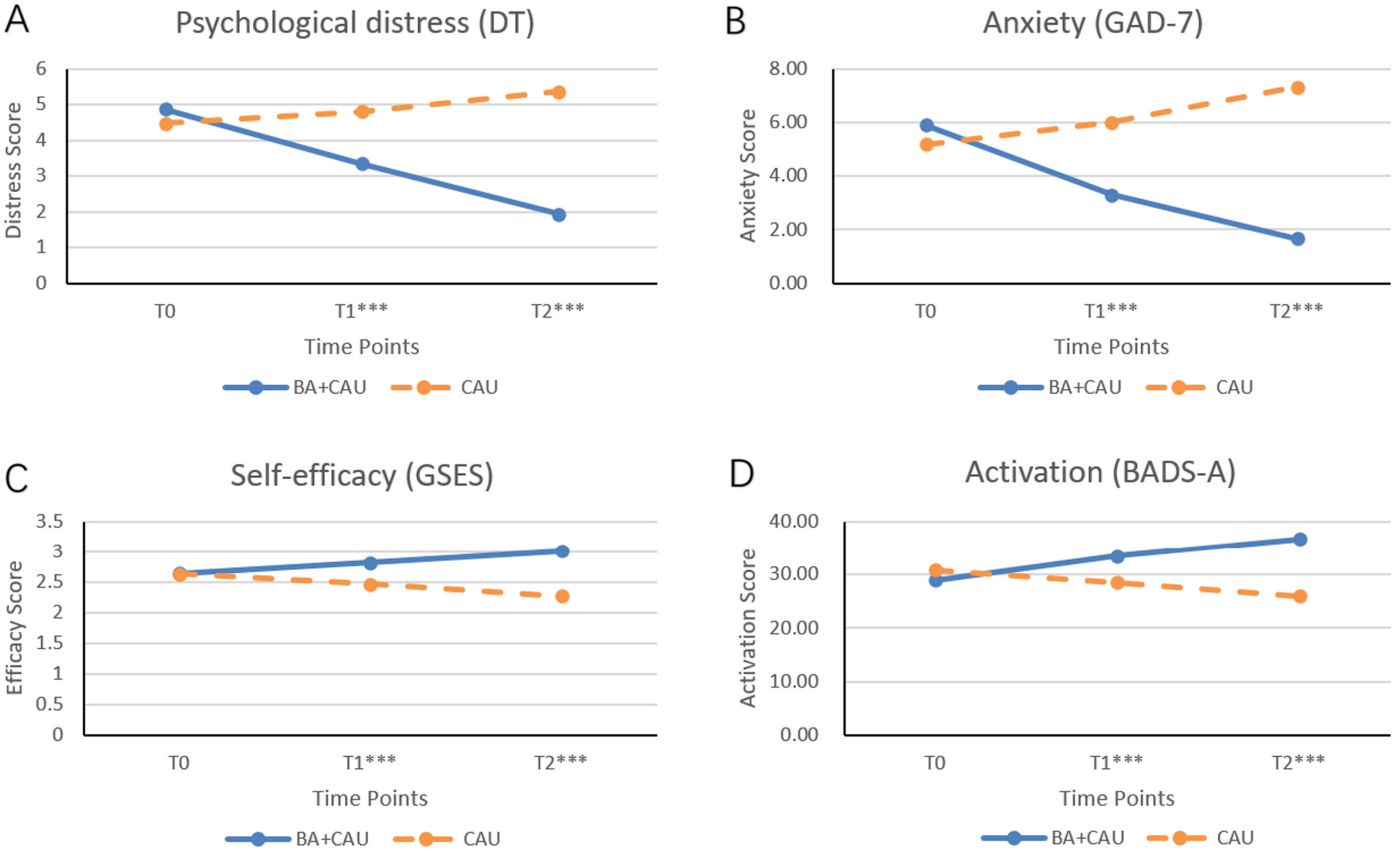
GEE analysis for the comparison of outcomes. ***p < 0.001.

Table 2 and Figure 3 show the results of GEE analysis. In comparison to the CAU group, the BA+CAU group exhibited significant improvements in psychological distress (time‐by‐group interaction, T1: *β* = −1.857, *p* < 0.001; T2: *β* = −3.786, *p* < 0.001) and anxiety (time‐by‐group interaction, T1: *β* = −3.456, *p* < 0.001; T2: *β* = −6.339, *p* < 0.001) at T1 and T2 assessments. Moreover, their self‐efficacy (time‐by‐group interaction, T1: *β* = 0.366, *p* < 0.001; T2: *β* = 0.749, *p* < 0.001) and activation (T1: *β* = 6.770, *p* < 0.001; T2: *β* = 12.522, *p* < 0.001) demonstrated notable enhancements at the end of T1 and T2 evaluations.

**TABLE 2:** Analysis of generalized estimating equation (GEE) for the comparison of outcomes.

Variable	*β*	95% CI	SE	Wald's *χ* ^2^	*p*
Psychological distress‐DT					
Intercept	4.478	4.235, 4.721	0.124	1304.075	< 0.001
Group (exp)^b^	0.393	−0.014, 0.800	0.208	3.586	0.058
Time (T1)^c^	0.327	0.029, 0.624	0.152	4.631	0.031
Time (T2)^c^	0.836	0.369, 1.304	0.239	12.292	< 0.001
Group (exp)*time (T1)^a^	−1.857	−2.248, −1.467	0.199	86.952	< 0.001
Group (exp)*time (T2)^a^	−3.786	−4.333, −3.238	0.279	183.681	< 0.001
Anxiety‐GAD‐7					
Intercept	5.188	4.212, 6.164	0.498	108.563	< 0.001
Group (exp)^b^	0.712	−0.704, 2.128	0.722	0.970	0.325
Time (T1)^c^	0.775	0.080, 1.470	0.355	4.775	0.029
Time (T2)^c^	2.052	1.086, 3.018	0.493	17.334	< 0.001
Group (exp)*time (T1)^a^	−3.456	−4.342, −2.571	0.452	58.536	< 0.001
Group (exp)*time (T2)^a^	−6.339	−7.559, −5.119	0.623	103.648	< 0.001
Self‐efficacy‐GSES					
Intercept	2.635	2.509, 2.760	0.064	1698.723	< 0.001
Group (exp)^b^	0.004	−0.164, 0.172	0.086	0.002	0.965
Time (T1)^c^	−0.184	−0.275, −0.093	0.047	15.620	< 0.001
Time (T2)^b^	−0.368	−0.485, −0.250	0.060	37.719	< 0.001
Group (exp)*time (T1)^a^	0.366	0.256, 0.475	0.056	42.892	< 0.001
Group (exp)*time (T2)^a^	0.749	0.609, 0.889	0.072	109.322	< 0.001
Activation‐BADS‐A					
Intercept	30.754	29.036, 32.471	0.876	1231.369	< 0.001
Group (exp)^b^	−1.854	−4.332, 0.625	1.265	2.149	0.143
Time (T1)^c^	−2.140	−3.090, −1.190	0.485	19.480	< 0.001
Time (T2)^c^	−4.683	−6.224, −3.141	0.786	35.462	< 0.001
Group (exp)*time (T1)^a^	6.770	5.552, 7.989	0.622	118.538	< 0.001
Group (exp)*time (T2)^a^	12.522	10.565, 14.479	0.999	157.266	< 0.001

Abbreviations: BADS‐A, the activation subscale of Behavioral Activation for Depression Scale; CI, confidence interval; DT, Psychological Distress Thermometer; exp., experimental; GAD‐7, Generalized Anxiety Disorder 7‐item scale; GEE, generalized estimating equation; GSES, General Self‐Efficacy Scale; SE, standard error.

^a^ Reference is group (control) × time (T0).

^b^ Reference is control group.

^c^ Reference is time (T0).

The findings of the stratified analyses (esophageal cancer and gastric cancer) are presented in Data S2. Table S1 show the results of GEE analysis for esophageal cancer patients. Consistent with the overall analysis findings, the time‐by‐group interaction terms of psychological distress (T1: *β* = −2.149, *p* < 0.001; T2: *β* = −4.131, *p* < 0.001), anxiety (T1: *β* = −3.019, *p* < 0.001; T2: *β* = −6.186, *p* < 0.001), self‐efficacy (T1: *β* = 0.373, *p* < 0.001; T2: *β* = 0.793, *p* < 0.001), and activation (T1: *β* = 7.128, *p* < 0.001; T2: *β* = 13.364, *p* < 0.001) exhibited significant improvements at T1 and T2 in the BA+CAU group compared to the CAU group. Similarly, the time‐by‐group interaction terms of the psychological distress (T1: *β* = −1.440, *p* < 0.001; T2: *β* = −3.276, *p* < 0.001), anxiety (T1: *β* = −4.135, *p* < 0.001; T2: *β* = −6.514, *p* < 0.001), self‐efficacy (T1: *β* = 0.387, *p* < 0.001; T2: *β* = 0.724, *p* < 0.001), and activation (T1: *β* = 6.512, *p* < 0.001; T2: *β* = 11.306, *p* < 0.001) of gastric cancer patients exhibited significant improvements at both T1 and T2 time points in the BA+CAU group compared to the CAU group (Table S2).

Based on the overall and subgroup analyses, it is evident that BA (directly) effectively ameliorates psychological distress and anxiety symptoms in patients with esophageal cancer and gastric cancer, both across the entire population and within specific cancer subtypes. Thus, scientific question 1 has been addressed.

### 3.3 Mediation Models

Before the mediation effect analysis, correlational analyses among psychological distress, anxiety, self‐efficacy and activation measured in our study were conducted to identify possible candidates for mediation effect analysis. Details are presented in Table 3. The results indicated activation that was positively correlated with self‐efficacy and negatively correlated with psychological distress and anxiety symptoms. At the same time, self‐efficacy was negatively correlated with psychological distress and anxiety symptoms.

**TABLE 3:** Correlational analyses of outcomes.

	DT	GAD‐7	GSES	BADS‐A
DT	1			
GAD‐7	0.742**	1		
GSES	−0.723**	−0.679**	1	
BADS‐A	−0.853**	−0.913**	0.745**	1

Abbreviations: BADS‐A, the activation subscale of Behavioral Activation for Depression Scale; DT, Psychological Distress Thermometer; GAD‐7, Generalized Anxiety Disorder 7‐item scale; GSES, General Self‐Efficacy Scale.

** *p* < 0.01.

This finding demonstrates that, in addition to BA, self‐efficacy serves as a protective factor against psychological distress. Furthermore, the GEE analysis presented above indicates that BA can effectively reduce psychological distress while simultaneously enhancing patients' self‐efficacy.

Based on these findings, literature review, and scientific question 2, we used self‐efficacy as the mediator variable, with activation as the independent variable and psychological distress as the dependent variable to build a mediation model.

**FIGURE 4:**
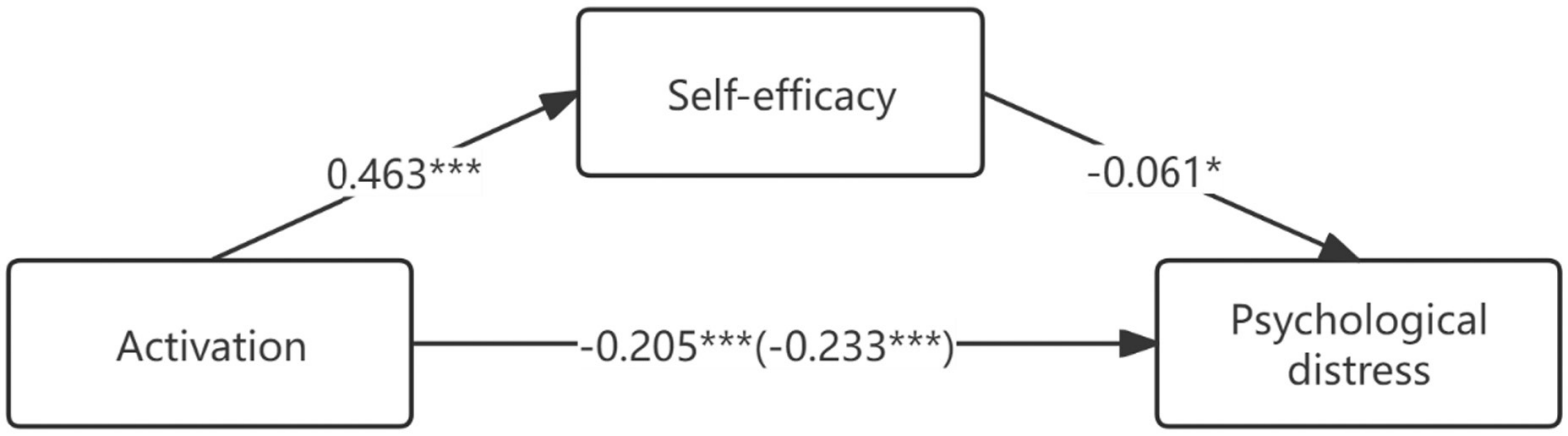
Meditating effect of self‐efficacy in activation. *p < 0.05, **p < 0.01, ***p < 0.001.

Detailed parameters of the mediation model of self‐efficacy between activation and psychological distress are shown in Table 4. All socio‐demographic and clinical factors were set as covariates. The results demonstrated that activation was negatively related to psychological distress (*B* = −0.233, *t* = −18.031, *p* < 0.001) and positively related to self‐efficacy (*B* = 0.463, t = 11.097, *p* < 0.001). The mediation test confirmed that self‐efficacy mediated the relationship between activation and psychological distress (*B* = −0.061, *t* = −2.210, *p* = 0.029). After controlling for the effect of self‐efficacy, the direct coefficient of activation on psychological distress was changed (*B* = −0.205, *t* = −11.355, *p* < 0.001). The mediation effect of self‐efficacy was confirmed by the coefficient change of activation. Figure 4 depicts the output model for the mediation effect of self‐efficacy between activation and psychological distress.

**TABLE 4:** Mediation model of self‐efficacy in activation and psychological distress.

	Psychological distress		Psychological distress		Self‐efficacy	
	*B*	*T*	*B*	*T*	*B*	*T*
Covariates						
Gender	−0.043	−0.145	−0.114	−0.386	1.177	1.229
Age	−0.007	−0.559	−0.008	−0.631	0.016	0.409
Work	−0.355	−1.993	−0.400	−2.222*	0.729	1.257
Education	0.107	0.825	0.146	1.124	−0.647	−1.540
Marriage	−0.544	−1.408	−0.507	−1.294	−0.602	−0.476
Caregiver	0.021	0.169	0.042	0.328	−0.335	−0.819
KPS	0.003	0.127	0.002	0.094	0.011	0.158
Primary tumor site	−0.077	−0.383	−0.087	−0.426	0.163	0.248
Tumor stage	−0.029	−0.197	−0.019	−0.126	−0.168	−0.345
Histology	0.056	0.251	0.072	0.318	−0.264	−0.361
Current treatment	−0.117	−1.566	−0.137	−1.814	0.322	1.326
BMI	−0.015	−0.389	−0.010	−0.262	−0.078	−0.614
Activation	−0.205	−11.355***	−0.233	−18.031***	0.463	11.097***
Self‐efficacy	−0.061	−2.210*				
*R* ^2^	0.756		0.746		0.532	
*F*	26.818***		27.626***		10.653***	

Abbreviations: *B*, regression coefficient; *T*, T statistic for coefficient; *R* ^2^, coefficient of determination for the model fitting; *F*, F statistic for the total regression model; KPS, Karnofsky performance status.

* *p* < 0.05.

*** *p* < 0.001.

As per the model, activation exhibit a negative correlation with psychological distress and a positive correlation with self‐efficacy, wherein self‐efficacy acts as a mediator between activation and psychological distress. Consequently, BA can (indirectly) alleviate psychological distress by enhancing self‐efficacy, thereby addressing scientific question 2.

## 4 Discussion

The findings of this experiment demonstrate that BA not only directly alleviates psychological distress and specific anxiety symptoms in patients with esophageal and gastric cancer but also indirectly mitigates psychological distress by enhancing patients' self‐efficacy. To ensure internal validity of these findings, we used various control mechanisms such as randomization, stratified analyses and covariate adjustment. Moreover, we used established scales with demonstrated high reliability and validity, thereby ensuring the robustness of our findings. Considering the demographics and health status of our sample, the generalizability of the findings could extend to similar populations, particularly elderly cancer patients experiencing high levels of psychological distress. However, these results should be cautiously applied to different age groups or types of cancer, as specific behavioral patterns and psychological experiences may vary.

The BA intervention demonstrated superiority over usual care, as evidenced by the GEE analysis which revealed a decline in general psychological distress and an improvement in self‐efficacy within the combination group, while these factors deteriorated over time among individuals receiving usual care. The findings of our overall analysis are further supported by stratified analyses. Our study demonstrate that BA can directly alleviate psychological distress and anxiety symptoms among patients diagnosed with esophageal cancer and/or gastric cancer (Question 1). This finding bears some resemblance to the conclusions drawn in two studies conducted by Gonzalez‐Fernandez et al. and Hirayama et al., which revealed that BA effectively alleviated negative emotions associated with depression and anxiety among cancer patients [36, 37]. However, it is worth noting that their investigations primarily focused on middle‐aged breast cancer patients, whereas our study predominantly concentrated on elderly individuals (66.04 ± 10.02 years old) diagnosed with esophageal and gastric cancer. Furthermore, our primary measure of interest was psychological distress rather than specific psychological symptoms. To the best of our knowledge, this study represents the first attempt to investigate whether BA can alleviate overall psychological distress among cancer patients.

Meanwhile, this study demonstrates the viability of BA interventions even when administered by primary healthcare professionals without specialized training. Our BA trainers have not received professional or systematic training. A large randomized controlled non‐inferiority trial has demonstrated that BA is equally effective as CBT, while being more feasible to implement and not requiring highly trained professionals for effective psychotherapy [38]. The external validity of this approach is promising, suggesting that BA could be adapted for clinical settings across different regions with varying levels of healthcare resources. This ensures that our findings are relevant and applicable in an international context, particularly in developing countries where access to specialized mental health professionals may be limited. This highlights the significant clinical potential of BA.

Regarding the intervention design, we used a decentralized randomized controlled trial (DCT). The integration of BA with both offline and online approaches (primarily remote online methods such as telephone or video conferencing by WeChat) can effectively alleviate psychological distress and anxiety symptoms among patients diagnosed with esophageal cancer and gastric cancer. Conducting electronically‐based DCT can effectively mitigate barriers to patient participation, facilitating increased interaction between patients and experimenters without being constrained by time or space limitations [39]. Given the flexibility and accessibility of these methods, the findings are likely to be applicable in various international contexts, where telemedicine is on the rise. Countries with different healthcare infrastructures can adopt these strategies to optimize patient outcomes. Consequently, this approach enhances patient adherence while alleviating the burden on healthcare professionals and patients to engage in clinical trials [13]. Moreover, our study achieved a commendable dropout rate of only 2.2%, further bolstering the reliability of our experimental findings.

In fact, BA was initially developed for addressing depression symptoms or major depression in the general population [40]. However, it possesses distinctive characteristics such as simplicity, structure, and flexibility of implementation. These features make it suitable for application in psychological interventions for cancer patients who face challenges related to open behavior and increasing life problems [41]. Previous research has demonstrated its effectiveness when applied to the psychological intervention of cancer patients [11, 13, 14]. However, the limited number of these studies necessitates further validation of the efficacy of BA in larger and more diverse cohorts of cancer patients. This study represents the first attempt to apply BA specifically to address psychological distress and anxiety symptoms in patients with esophageal and gastric cancer.

Diverging from external psychological interventions, self‐efficacy represents individuals' internal personal belief in their capacity to organize and execute the necessary actions to attain specific achievement goals [42]. It serves as a protective factor against negative emotions. Among cancer patients, self‐efficacy plays a crucial role in their psychological adjustment to the significant impact of cancer [43]. Individuals with higher levels of self‐efficacy exhibit a more positive attitude toward managing the adverse effects of cancer treatment and mitigating negative emotions such as depression and anxiety. For instance, among rectal cancer patients who have undergone permanent colostomy, those with high self‐efficacy demonstrate fewer symptoms of anxiety [44]. Furthermore, a higher level of self‐efficacy at the baseline was found to be significantly associated with improved emotional well‐being 12 months later in breast cancer patients [45]. Additionally, in head and neck cancer patients experiencing facial disfigurement, self‐efficacy plays a crucial role in mitigating the impact of social distress on emotional well‐being [46].

Similarly, we observed a significant correlation between high levels of self‐efficacy and reduced anxiety symptoms among patients diagnosed with esophageal and gastric cancer. But differently, a significant correlation between high levels of self‐efficacy and reduced overall psychological distress was also observed. Furthermore, BA interventions not only ameliorated psychological distress but also enhanced the self‐efficacy of these individuals. These findings suggest that self‐efficacy might serve as a mediator in the process of BA. Therefore, we have developed a mediation model to investigate the potential mediating role of self‐efficacy between BA and psychological distress. The results from our model demonstrate that self‐efficacy indeed plays a significant mediating role in this relationship. The impact of BA on psychological distress is partially mediated by self‐efficacy. This finding aligns with international research conducted by Annesi et al. [47] and Au et al. [48] that emphasizes the importance of self‐efficacy in mental health interventions, making our results relevant across different cultural backgrounds. However, there exist several distinctions between our study and these previous works. The former primarily focused on emotional eating and involved obese women, whereas the latter encompassed family caregivers of dementia patients. Furthermore, both studies used more specific measures of self‐efficacy (e.g., self‐efficacy to control eating and self‐efficacy to manage disturbed thoughts) rather than assessing general self‐efficacy. While we have discovered for the first time that general self‐efficacy can serve as a mediator between BA and psychological distress in cancer patients (Question 2).

Notably, there is currently a dearth of studies examining the mediating effect size of self‐efficacy in psychological interventions. Consequently, further research is warranted to explore whether self‐efficacy can serve as a mediator in other psychological interventions. Our study stands as the pioneering effort to elucidate the underlying psychological mechanism through which BA influences psychological distress among cancer patients. However, it remains an open question whether additional mechanisms exist for BA and other psychological interventions.

### 4.1 Limitations

Our study also has certain limitations. First, our study is a single‐blind randomized controlled trial conducted in a solitary center, which may impose limitations on the generalizability of our findings. In future investigations, it would be imperative to conduct multi‐center double‐blind or even triple‐blind randomized controlled trials to corroborate our conclusions. Additionally, international collaborations could be explored to see how our findings hold in different cultural contexts and healthcare settings, enhancing the validity and reliability of the interventions globally. Second, due to scientific question and the limited sample size of building another mediation model, we established a mediating model with psychological distress as the dependent variable, but did not establish another one with anxiety as the dependent variable. However, psychological distress encompasses a broad spectrum of physical, familial, emotional, spiritual/religious, and physiological issues [1]. It also encompasses a wide range of commonly experienced negative emotional symptoms, such as depression, anxiety and fear. Naturally, it is imperative to validate whether BA can influence specific negative emotional symptoms through the mediation of self‐efficacy in studies with adequate sample sizes such as large‐scale longitudinal cohort studies or cross‐sectional investigations. Third, it is necessary to validate and explore other pathways through which BA affects psychological distress in cancer population, as this study specifically focused on self‐efficacy. In summary, future research should conduct larger‐scale, multi‐center, multi‐cultural and multicancer type RCT with diverse research pathways to comprehensively explore the role of BA in psychological distress and specific negative emotional symptoms of cancer populations. This will facilitate its broader adoption and application.

### 4.2 Clinical Implications

Our study may offer a more efficacious approach for oncologists, nurses, or healthcare professionals lacking formal psychological training to alleviate psychological distress experienced by cancer patients. Given the global rise in cancer cases and disparities in mental health care provision, our findings have important implications for international healthcare practices. BA, a simple and flexible psychosocial intervention, can be a viable option in both high‐resource and low‐resource settings. Furthermore, psychosocial interventions such as BA should emphasize enhancing patients' self‐efficacy within the intervention process to maximize their effectiveness.

## 5 Conclusions

Behavioral activation not only directly alleviated psychological distress and anxiety symptoms in patients with esophageal cancer and gastric cancer but also indirectly mitigated psychological distress by enhancing self‐efficacy as a mediator. Our study demonstrates the broad potential application of behavioral activation, a simple and flexible psychosocial intervention for cancer patients, highlighting its efficacy in addressing emotional well‐being.

## Author Contributions

**Runze Huang:** data curation (equal), formal analysis (lead), methodology (lead), resources (lead), software (lead), visualization (equal), writing – original draft (lead), writing – review and editing (equal). **Anlong Li:** conceptualization (lead), investigation (lead), methodology (equal), project administration (equal), supervision (equal), validation (equal), writing – review and editing (equal). **Han Ge:** formal analysis (equal), investigation (equal), project administration (equal), supervision (equal), validation (equal), visualization (equal), writing – review and editing (equal). **Lijun Liu:** project administration (supporting), supervision (supporting), validation (supporting), writing – review and editing (supporting). **Ling Cheng:** validation (equal), writing – original draft (equal), writing – review and editing (equal). **Mingjun Zhang:** supervision (equal), validation (equal), writing – review and editing (equal). **Huaidong Cheng:** project administration (equal), supervision (lead), writing – review and editing (equal).

## Ethics Statement

All procedures, including the informed consent process, were conducted in accordance with the ethical standards of the responsible committee on human experimentation (institutional and national) and with the Helsinki Declaration of 1975, as revised in 2000. Participation was strictly voluntary and all responses were anonymous. Besides, the research was approved by the Research Ethics Board at Anhui Medical University (Ethical number: 83242392).

## Consent

The authors have nothing to report.

## Conflicts of Interest

The authors declare no conflicts of interests.

## Supporting information


Data S1.

Data S2.

## Data Availability

The datasets used and/or analyzed in this study can be handed out in anonymised form. Please contact the author Runze Huang (18756909618@163.com) in this regard.
