# Supplementary material for: Impacts and Pathways of Behavioral Activation on Psychological Distress Among Patients Diagnosed With Esophageal and Gastric Cancer in China: A Randomized Controlled Trial
Source: Cancer Med. 2024 Oct 15;13(19):e70314. doi: 10.1002/cam4.70314 (PMC11475026; doi:10.1002/cam4.70314)
Supplement: Supplementary file 1 — Data S1. [file CAM4-13-e70314-s002.docx]

**English version of BA intervention program (8 sessions, once a week)**

| Sessions | Key elements | Weekly Assignments |
| --- | --- | --- |
| Session1 (Special session)  Offline intervention | 1.Discussion of Depression  2.Introduction to Treatment Rationale. What about stressful life events and loss in your life?  3.Introduction to Daily Monitoring: (1) Importance and enjoyment (or meaning and happiness) ratings. (2) When should you complete the Daily Monitoring Form?  4.Important Points About the Structure of This Treatment | Complete Daily Monitoring Form |
| Session 2  (Special session)  Via telephone or WeChat | 1.Daily Monitoring: Review Assignment, Troubleshooting  2.Treatment Rationale: Review  3.Complete Life Areas, Values, Activities Inventory (Appendix) | 1. Complete Daily Monitoring  2. Review and edit Life Areas, Values, and Activities Inventory |
| Session 3  (Special session)  Via telephone or WeChat | 1.Daily Monitoring: Review Assignment  2.Life Areas, Values, and Activities Inventory: Review assignment  3.Activity Selection and Ranking | 1.Daily monitoring  2.Continue to review and edit Life Areas, Values, and Activities Inventory  3. Review and edit activity selection and ranking |
| Session 4  (Special session)  Via telephone or WeChat | 1.Daily Monitoring: Review Assignment  2.Daily Monitoring with Activity Planning | Daily Monitoring with activity planning for upcoming week |
| Session 5  (Special session)  Via telephone or WeChat | 1.Daily Monitoring With Activity Planning: Review Assignment  2.Contract: Ensure the timely completion of activities through effective collaboration with other people  3.Daily Monitoring With Activity Planning for the Upcoming Week | 1.Daily monitoring with activity planning for the upcoming week  2.Continue adding/editing contracts |
| Session 6  (Maintenance  Session) Via telephone or WeChat | 1.Daily Monitoring With Activity Planning: Review Assignment  2.Life Areas, Values, and Activities Inventory: Concept Review and Edit  3.Daily Monitoring With Activity Planning for the Upcoming Week | 1.Daily monitoring with activity planning for the upcoming week  2.Continue adding/editing Contracts |
| Session 7  (Maintenance  Session) Via telephone or WeChat | 1.Daily Monitoring With Activity Planning: Review Assignment  2.Life Areas, Values, and Activities Inventory: Concept Review and Edit  3.Daily Monitoring With Activity Planning for the Upcoming Week | 1.Daily monitoring with activity planning for the upcoming week  2.Continue adding/editing contracts |
| Session 8  (Maintenance  Session) Via telephone or WeChat | 1.Daily Monitoring With Activity Planning: Review Assignment  2.Daily Monitoring With Activity Planning for the Upcoming Week  3.Preparing for the End of Treatment | 1.Daily monitoring with activity planning for the upcoming week  2.Continue adding/editing contracts |

**Appendix**

*Moving From Life Areas and Values to Activities*

Life area (1/5): Relationships

Value: Being a loving parent

Activity:Tell my child I love them every day

Activity: Make a special breakfast for my child on Saturday

Activity: Pick up my child from school promptly each day

Value: Being an attentive and caring friend

Activity: Call my friend once per week

Activity:Text my friend

Activity:Ask my friend about his or her week

Value: Caring for the needs of your spouse

Activity: Make special plans with spouse

Activity:Tell spouse I love them every day

Activity: Buy my partner a surprise gift

Life area (2/5): Education/career

Value: Get more formal education

Activity:Ask a friend for advice about school

Activity:Write out a plan for enrolling in school

Value: Learn new skills for work

Activity:Ask someone at work to teach me a new skill

Activity:Take a class

Value: Be knowledgeable about the world around you

Activity: Read the newspaper everyday

Activity:Talk about current events to a stranger

Value: Improve your job performance and satisfaction

Activity: Set a work-related goal

Activity: Read a book about my profession

Value: Find a new job that fits with your skills and interests

Activity: Look at job advertisements

Activity:Talk to someone who has a job available

Life area (3/5): Recreation/interests

Value: Being active

Activity: Go to the park with my son

Activity:Take a walk outside

Activity: Play football on Saturday

Value: Being artistic and creative

Activity: Knit

Activity: Make crafts

Value: Making a sacrifice for something you believe in

Activity: Start a petition

Activity: Spend time helping at my place of worship

Value: Helping others less fortunate or who need help

Activity: Donate clothes

Activity: Spend 30 min helping an elderly person

Value: Showing a commitment to your country

Activity: Vote in an election

Activity: Volunteer for a campaign

Life area (4/5): Mind/body/spirituality

Value: Being physically healthy

Activity: Go to a doctor for a physical/check-up

Activity: Eat fruit everyday

Activity: Take my medication as prescribed

Value: Talking to someone about your problems and feelings

Activity: Ask someone to lunch to talk

Activity: Make an appointment with a therapist

Activity: Write in a journal

Value: Developing your religious/spiritual views

Activity: Talk with a religious figure

Activity: Talk to others with religious beliefs you are interested in

Value: Living a spiritual life

Activity: Pray everyday

Activity: Read my religious material

Activity: Attend a religious service

Value: Being tolerant, nonjudgmental, accepting of others differences

Activity: Talk to someone with a different background

Activity: Read a book about a different culture

Life area (5/5): Daily responsibilities

Value: Being someone others can depend on

Activity: Arrive at work on time

Activity: Offer to help someone who is very busy

Activity: Repay a debt

Value: Taking care of your belongings

Activity: Complete a much-needed household repair

Activity: Wash your clothes and shoes

Activity: Clean the house

Value: Being organized

Activity: Review my days activities the night before

Activity: Use a calendar to record dates and meetings

Activity: Develop a filing system for important paperwork

**行为激活干预课程（中文版）8个疗程，每周一次**

**Chinese version of BA intervention program (8 sessions, once a week)**

| **课程** | **核心要点** | **每周任务** |
| --- | --- | --- |
| Session 1（特殊课程）  线下进行 | 1.抑郁的讨论  2.治疗原理的介绍·关于你生活中的压力事件和失落？  3.日常监测入门：快乐和意义性评级，你应该什么时候完成每日监测表？  4.关于这种治疗结构的要点 | 填写每日监测表 |
| Session 2  （特殊课程）  电话或微信 | 1.日常监测：评审作业·故障排除  2.治疗原理：评审  3.完整的生命领域、价值和活动清单（附录） | 1.填写每日监测表格  2.审查和编辑生活领域、价值和活动清单 |
| Session 3  （特殊课程）  电话或微信 | 1.日常监控：评审作业  2.生活领域、价值和活动清单：评审作业  3.活动选择和排名 | 1. 日常监测  2. 继续审查和编辑生活领域、价值观和活动清单  3. 审查和编辑活动选择和排名 |
| Session 4  （特殊课程）  电话或微信 | 1.日常监控：审核作业  2.日常监控和计划 | 每日监控以及下周的活动计划 |
| Session 5  （特殊课程）  电话或微信 | 1.日常监控与活动计划：审核作业  2.合同：通过与他人合作督促确保活动的完成  3. 每日监控与下周活动计划 | 1. 每日监控以及下周的活动计划  2. 继续添加/编辑合同 |
| Session 6  （维持课程）  电话或微信 | 1.每日监控与活动计划：审查作业  2.生活领域、价值观和活动清单：概念审查和编辑  3. 每日监控与下周活动计划 | 1. 日常监控以及下周的活动计划  2. 继续添加/编辑合同 |
| Session 7  （维持课程）  电话或微信 | 1.每日监控与活动计划：审查作业  2.生活领域、价值观和活动清单：概念审查和编辑  3. 每日监控与下周活动计划 | 1. 日常监控以及下周的活动计划  2. 继续添加/编辑合同 |
| Session 8  （维持课程）  电话或微信 | 1.每日监测和活动计划：审核作业  2.每日监测和下周活动计划  3. 为治疗结束做准备 | 1. 日常监控以及下周的活动计划  2. 继续添加/编辑合同 |

附：完整的生命领域、价值和活动清单

**生活领域（1/5）：人际关系**

**价值：做一个慈爱的父母**

活动：告诉我的孩子，我每天都爱他们

活动：星期六为我的孩子做一顿特别的早餐

活动：每天及时接我的孩子

**价值：成为一个细心和关心的朋友**

活动：每周给我的朋友打电话一次

活动：发短信给我的朋友

活动：问我的朋友关于他或她的一周

**价值：照顾你的配偶的需求**

活动：与配偶一起制定特殊计划

活动：告诉配偶我每天都爱他们

活动：给我的伴侣买一份令人惊喜的礼物

**生活领域（2/5）：教育程度/职业生涯**

**价值：接受更正规的教育**

活动：向朋友关于学校的建议

活动：写出一个入学的计划

**价值：学习工作中的新技能**

活动：请工作中的人教我一种新技能

活动：上课

**价值：了解你周围的世界**

活动：每天阅读报纸

活动：向陌生人谈论时事

**价值：提高你的工作表现和满意度**

活动：设定一个与工作相关的目标

活动：读一本关于我的职业的书

**价值：找一份符合你的技能和兴趣的新工作**

活动：看看招聘广告

活动：与有工作机会的人交谈

**生活领域（3/5）：娱乐/兴趣**

**价值：处于活动状态**

活动：和我的儿子一起去公园

活动：外出散步

活动：周六踢足球

**价值：具有艺术性和创造性**

活动：针织

活动：制作工艺

**价值：为你所相信的东西做出牺牲**

活动：启动请愿书

活动：花时间在我的礼拜场所帮忙

**价值：帮助那些不幸的人或需要帮助的人**

活动：捐赠衣物

活动：花30个min来帮助一个老人

**价值：表示对你的国家的承诺**

活动：在选举中投票

活动：自愿参加一个竞选活动

**生活领域（4/5）：身心、精神方面**

**价值：身体健康**

活动：去医生做体检

活动：每天吃水果

活动：按规定服药

**价值：与别人谈论你的问题和感受**

活动：请某人吃午餐交谈

活动：与治疗师进行预约

活动：在日记帐中写入

**价值：发展你的宗教/精神观点**

活动：与一个宗教人物交谈

活动：与那些带有你对价值感兴趣的宗教信仰的人交谈：过一种精神生活

活动：每天祈祷

活动：阅读我的宗教材料

活动：参加一个宗教仪式

活动：与有不同背景的人交谈

活动：读一本关于另一种文化的书

**生活区域（5/5）：日常责任**

**价值：成为别人可以依赖的人**

活动：按时到达工作

活动：主动帮助那些非常忙的人

活动：偿还债务

**价值：照顾好你的随身物品**

活动：完成一个急需的家庭维修

活动：洗好衣服和鞋子

活动：打扫房间

**价值：正在组织**

活动：回顾一下我白天在前一天晚上的活动

活动：使用日历来记录日期和会议

活动：为重要的文书工作建立一个归档系统
