# Supplementary material for: Impacts and Pathways of Behavioral Activation on Psychological Distress Among Patients Diagnosed With Esophageal and Gastric Cancer in China: A Randomized Controlled Trial
Source: Cancer Med. 2024 Oct 15;13(19):e70314. doi: 10.1002/cam4.70314 (PMC11475026; doi:10.1002/cam4.70314)
Supplement: Supplementary file 2 — Data S2. [file CAM4-13-e70314-s001.docx]

**Table S1** Analysis of GEE for the comparison of outcomes for esophageal cancer patients

| Variable | β | 95% CI | SE | Wald’s χ2 | P-value |
| --- | --- | --- | --- | --- | --- |
| **Psychological distress-DT** |  |  |  |  |  |
| Intercept | 4.303 | 3.995, 4.611 | 0.157 | 747.654 | <0.001 |
| Group (exp)† | 0.671 | 0.110, 1.232 | 0.286 | 5.500 | 0.019 |
| Time (T1)‡ | 0.636 | 0.253, 1.020 | 0.196 | 10.592 | 0.001 |
| Time (T2)‡ | 1.182 | 0.492, 1.872 | 0.352 | 11.274 | 0.001 |
| Group (exp)*time (T1)§ | -2.149 | -2.690, -1.608 | 0.276 | 60.618 | <0.001 |
| Group (exp)*time (T2)§ | -4.131 | -4.906, -3.355 | 0.396 | 108.886 | <0.001 |
| **Anxiety-GAD-7** |  |  |  |  |  |
| Intercept | 5.091 | 3.887, 6.295 | 0.614 | 68.717 | <0.001 |
| Group (exp)† | 0.807 | -0.961, 2.574 | 0.902 | 0.800 | 0.371 |
| Time (T1)‡ | 0.788 | -0.223, 1.798 | 0.516 | 2.335 | 0.127 |
| Time (T2)‡ | 2.212 | 0.871, 3.553 | 0.684 | 10.459 | 0.001 |
| Group (exp)*time (T1)§ | -3.019 | -4.234, -1.803 | 0.620 | 23.680 | <0.001 |
| Group (exp)*time (T2)§ | -6.186 | -7.828, -4.545 | 0.837 | 54.577 | <0.001 |
| **Self-efficacy-GSES** |  |  |  |  |  |
| Intercept | 2.727 | 2.557, 2.897 | 0.087 | 989.560 | <0.001 |
| Group (exp)† | -0.094 | -0.318, 0.130 | 0.114 | 0.675 | 0.411 |
| Time (T1)‡ | -0.194 | -0.328, -0.060 | 0.068 | 8.065 | 0.005 |
| Time (T2)‡ | -0.418 | -0.593, -0.244 | 0.089 | 22.070 | <0.001 |
| Group (exp)*time (T1)§ | 0.373 | 0.215, 0.532 | 0.081 | 21.430 | <0.001 |
| Group (exp)*time (T2)§ | 0.793 | 0.592, 0.993 | 0.102 | 60.213 | <0.001 |
| **Activation-****BADS-A** |  |  |  |  |  |
| Intercept | 31.303 | 29.045, 33.561 | 1.152 | 738.485 | <0.001 |
| Group (exp)† | -2.534 | -5.758, 0.691 | 1.645 | 2.372 | 0.124 |
| Time (T1)‡ | -2.667 | -3.959, -1.375 | 0.659 | 16.361 | <0.001 |
| Time (T2)‡ | -5.697 | -7.846, -3.548 | 1.096 | 27.001 | <0.001 |
| Group (exp)*time (T1)§ | 7.128 | 5.527, 8.729 | 0.817 | 76.157 | <0.001 |
| Group (exp)*time (T2)§ | 13.364 | 10.702, 16.025 | 1.358 | 96.842 | <0.001 |

**Table S2** Analysis of GEE for the comparison of outcomes for gastric cancer patients

| Variable | β | 95% CI | SE | Wald’s χ2 | P-value |
| --- | --- | --- | --- | --- | --- |
| **Psychological distress-DT** |  |  |  |  |  |
| Intercept | 4.593 | 4.233, 4.952 | 0.183 | 627.118 | <0.001 |
| Group (exp)† | 0.157 | -0.464, 0.779 | 0.317 | 0.247 | 0.619 |
| Time (T1)‡ | -0.067 | -0.597, 0.464 | 0.271 | 0.060 | 0.806 |
| Time (T2)‡ | 0.297 | -0.449, 1.044 | 0.381 | 0.609 | 0.435 |
| Group (exp)*time (T1)§ | -1.440 | -2.070, -0.811 | 0.321 | 20.120 | <0.001 |
| Group (exp)*time (T2)§ | -3.276 | -4.210, -2.342 | 0.476 | 47.291 | <0.001 |
| **Anxiety-GAD-7** |  |  |  |  |  |
| Intercept | 6.037 | 4.182, 7.892 | 0.946 | 40.690 | <0.001 |
| Group (exp)† | 0.088 | -2.567, 2.742 | 1.354 | 0.004 | 0.948 |
| Time (T1)‡ | 0.596 | -0.546, 1.737 | 0.583 | 1.046 | 0.306 |
| Time (T2)‡ | 1.490 | -0.075, 3.055 | 0.799 | 3.483 | 0.062 |
| Group (exp)*time (T1)§ | -4.135 | -5.664, -2.606 | 0.780 | 28.111 | <0.001 |
| Group (exp)*time (T2)§ | -6.514 | -8.596, -4.431 | 1.063 | 37.571 | <0.001 |
| **Self-efficacy-GSES** |  |  |  |  |  |
| Intercept | 2.544 | 2.361, 2.728 | 0.094 | 738.991 | <0.001 |
| Group (exp)† | 0.093 | -0.190, 0.376 | 0.144 | 0.416 | 0.519 |
| Time (T1)‡ | -0.186 | -0.341, -0.030 | 0.079 | 5.482 | 0.019 |
| Time (T2)‡ | -0.306 | -0.506, -0.106 | 0.102 | 9.006 | 0.003 |
| Group (exp)*time (T1)§ | 0.387 | 0.207, 0.568 | 0.092 | 17.678 | <0.001 |
| Group (exp)*time (T2)§ | 0.724 | 0.484, 0.963 | 0.122 | 35.030 | <0.001 |
| **Activation-BADS-A** |  |  |  |  |  |
| Intercept | 28.815 | 25.850, 31.780 | 1.513 | 362.864 | <0.001 |
| Group (exp)† | 0.310 | -4.052, 4.672 | 2.225 | 0.019 | 0.889 |
| Time (T1)‡ | -1.433 | -3.010, 0.144 | 0.805 | 3.170 | 0.075 |
| Time (T2)‡ | -2.978 | -5.278, -0.677 | 1.174 | 6.435 | 0.011 |
| Group (exp)*time (T1)§ | 6.512 | 4.370, 8.653 | 1.093 | 35.520 | <0.001 |
| Group (exp)*time (T2)§ | 11.306 | 8.148, 14.465 | 1.612 | 49.227 | <0.001 |
